# *Bacillus* spp.-Mediated Growth Promotion of Rice Seedlings and Suppression of Bacterial Blight Disease under Greenhouse Conditions

**DOI:** 10.3390/pathogens11111251

**Published:** 2022-10-28

**Authors:** Faheem Uddin Rajer, Muhammad Kaleem Samma, Qurban Ali, Waleed Ahmed Rajar, Huijun Wu, Waseem Raza, Yongli Xie, Hafiz Abdul Samad Tahir, Xuewen Gao

**Affiliations:** 1 Key Laboratory of Integrated Management of Crop Diseases and Pests, Ministry of Education, Department of Plant Pathology, College of Plant Protection, Nanjing Agricultural University, Nanjing 210095, China; 2 Department of Plant Pathology, Faculty of Crop Protection, Sindh Agriculture University, Tandojam 70060, Pakistan; 3 Department of Biosciences, Shaheed Zulfiqar Ali Bhutto Institute of Science and Technology, Karachi 75600, Pakistan; 4 Institute of Microbiology, University of Sindh, Jamshoro 76080, Pakistan; 5 Jiangsu Key Lab for Organic Solid Waste Utilization, Nanjing Agricultural University, Nanjing 210095, China; 6 State Key Laboratory of Plateau Ecology and Agriculture, Department of Grassland Science, College of Agriculture and Animal Husbandry, Qinghai University, Xining 810016, China; 7 Tobacco Research Institute, Pakistan Tobacco Board, Ministry of National Food Security and Research, Peshawar 25124, Pakistan

**Keywords:** *Bacillus* spp., broad-range antagonism, bacterial blight, biocontrol, growth promotion, lipopeptides

## Abstract

Rice (*Oryza sativa* L.) is a major cereal and staple food crop worldwide, and its growth and production are affected by several fungal and bacterial phytopathogens. Bacterial blight (BB) is one of the world’s most devastating rice diseases, caused by *Xanthomonas oryzae* pv. *oryzae* (*Xoo*). In the current study, *Bacillus atrophaeus* FA12 and *B. cabrialesii* FA26 were isolated from the rice rhizosphere and characterized as having broad-range antifungal and antibacterial activities against various phytopathogens, including *Xoo*. In addition, the selected strains were further evaluated for their potent rice growth promotion and suppression efficacy against BB under greenhouse conditions. The result shows that FA12 and FA26, applied as seed inoculants, significantly enhanced the vigor index of rice seedlings by 78.89% and 108.70%, respectively. Suppression efficacy against BB disease by FA12 and FA26 reached up to 59.74% and 54.70%, respectively, in pot experiments. Furthermore, MALDI-TOF MS analysis of selected strains revealed the masses ranged from *m*/*z* 1040 to 1540, representing that iturins and fengycin are the major antimicrobial compounds in the crude extracts, which might have beneficial roles in rice defence responses against BB. In conclusion, FA12 and FA26 possess broad-range antagonistic activity and have the capability to promote plant growth traits. More importantly, applying these strains has a high potential for implementing eco-friendly, cost-effective, and sustainable management practices for BB disease.

## 1. Introduction

Rice (*Oryza sativa* L.) is an important staple food crop for rice-growing countries in Asia, particularly China, where 30% of the world’s rice is cultivated [1]. However, due to a variety of biotic and abiotic stresses, the average rice production decreased its potential [2,3]. The common fungal phytopathogenic diseases such as rice blast caused by *Magnaporthe oryzae*, rice seedling blight caused by *Fusarium graminearum*, rice bakanae caused by *F. moniliforme*, and rice sheath blight caused by *Rhizoctonia solani*, and bacterial disease, for example, bacterial blight (BB) caused by *Xanthomonas oryzae* pv. *oryzae* (*Xoo*), cause significant crop losses and production damage [4,5]. BB disease is one of the major constraints in rice production and is widely distributed among all major rice-growing countries, including China [6]. Yield losses by BB at the maximum tillering stage range from 20–40% [7,8]. Using chemicals, resistant cultivars, and biocontrol agents are important measures for this disease [8]; however, chemicals may leave toxic effects in the environment, and breeding resistant genes has no long-lasting durability [7] in this fast-moving world. Biocontrol of plant diseases using antagonistic microbes is an eco-friendly and cost-effective substitute for crop protection [9,10]. Therefore, there is an increasing demand to search for new biocontrol agents (BCAs) with broad-range antagonistic activity against multiple phytopathogens [11,12]. Suppression of BB has been attempted with several microbial antagonists, including the strains of *Bacillus* spp. and *Pseudomonas* spp. [7,13].

*Bacillus* spp. are widely distributed plant-growth-promoting rhizobacteria (PGPR) in soil, forming robust endospores resistant to raised temperature and high concentrations of chemicals [14]. *Bacillus* spp. promote plant growth by enhancing seed emergence, biomass, and yield [15,16,17]. Mechanisms involved in the growth promotion of plants by PGPR include their synthesis of phytohormones such as cytokinin, indole-3-acetic acid (IAA), and gibberellin; the breakdown of plant-produced ethylene by bacterial production of 1-aminocyclopropane-1-carboxylate (ACC) deaminase [16]; and increased uptake of available minerals, phosphorus, and nitrogen in the soil [18,19]. Biocontrol mechanisms by *Bacillus* spp. include the production of antimicrobial compounds such as lipopeptides by the nonribosomal peptide synthetase pathway [20] and polyketides, etc., against several phytopathogens and induction of systemic resistance of the host plants [17,21,22] by eliciting the production of plant defensive compounds such as peroxidase and chitinase, exerting protective effects against phytopathogens [23]. Generally, an efficient in vitro screening strategy is an initial step required for newly isolated strains toward developing potential BCAs. During the screening, it is good to add top-ranked economically important fungal and/or bacterial phytopathogens [24,25] together with the target phytopathogens. In our screening, we included some of the important rice pathogens such as *M. oryzae*, *R. solani*, and *Xoo*. In addition, pathogens having a broader host range, such as *Sclerotinia sclerotiorum*, *F. oxysporum*, and *Phytophthora capsici*, were added. One of the major reasons behind failing potential antagonists selected based on in vitro screening under field conditions may be encountering several other pathogens in the same field [26].

In this study, we aimed at screening *Bacillus* strains isolated from the rice rhizosphere for their broad-range antagonism activity against multiple phytopathogens. The selected strains were also evaluated for their potential growth promotion of rice seedlings and effective suppression of BB disease under greenhouse conditions. Furthermore, matrix-assisted laser desorption ionization–time of flight mass spectrometry (MALDI-TOF MS) was used to characterize the antimicrobial lipopeptides produced by the selected antagonists.

## 2. Materials and Methods

### 2.1. Bacillus Strains and Their Growth Conditions

Soil samples were collected from the rhizosphere of rice grown in the vicinity of Sindh Agriculture University, Tandojam, Sindh, Pakistan. Five grams of each composite soil sample (dry weight) was suspended in 45 mL of sterile 0.9% NaCl solution (*w*/*v*) and mixed thoroughly on a rotatory shaker (200 rpm) for 30 min at 37 °C. To select for spore-forming bacteria (*Bacillus* spp.), samples were heat-shocked at 80 °C for 10 min [14,27] followed by 7-fold serial dilution and plated on Luria–Bertani (LB) medium. Plates were incubated at 37 °C overnight. The colonies were visually distinguished based on morphological characteristics typical of *Bacillus* spp., as described previously [27], and were streak-purified at least three times. Isolates were tentatively identified as *Bacillus* spp. and stored in LB broth with 30% glycerol at −80 °C for further use.

*Bacillus velezensis* FZB42, a Gram-positive model bacterium commercially used as a biofertilizer and biocontrol agent in agriculture [28], was considered as the reference strain. FZB42 was procured from frozen stock (−80 °C). All the strains were grown routinely on LB at 37 °C overnight.

### 2.2. Phytopathogens Used in the Study

Five fungal phytopathogens (*Sclerotinia sclerotiorum*, *Rhizoctonia solani*, *Fusarium oxysporum*, *Phytophthora capsici*, and *Magnaporthe oryzae*) and three phytopathogenic bacteria (*Erwinia amylovora*, *Clavibacter michiganensis* ssp. *sepedonicus* (*Cms*), and *Xanthomonas oryzae* pv. *oryzae* (*Xoo*)) were selected as test phytopathogens. All of the phytopathogens were procured from their respective laboratory culture stocks. *S. sclerotiorum* and *Xoo* were obtained from the Lab. of Plant Disease Control and Phytochemistry (College of Plant Protection, Nanjing Agricultural University (NJAU), Nanjing, China). *P. capsici*, *R. solani*, *F. oxysporum*, and *M. oryzae* were obtained from the Lab. of Plant–Phytophthora Interactions, NJAU, while, *E. amylovora* and *Cms* were provided by the Lab. of Plant Quarantine and Applied Immunology (NJAU). *S. sclerotiorum* and *F. oxysporum* were grown on potato dextrose agar (PDA) with a pH of 7.0. *R. solani* was also grown on PDA, however, pH was adjusted to 5.6 [29]. *P. capsici* and *M. oryzae* were grown on V8 juice agar [30] and on yeast extract glucose (YEG) medium (5 g of yeast extract, 10 g of D-glucose, and 15 g of agar per 1 L of MQ water, and the pH was adjusted to 7.0), respectively. These fungal phytopathogens were stored at 25 °C and were maintained axenically by subculturing onto their respective fresh media plates at regular intervals when their food supply was exhausted. At the same time, agar slants were also prepared and stored at 4 °C as working stocks. The phytopathogenic bacteria were grown routinely on nutrient agar (NA) medium at 28 °C for 3–5 days, as described previously [31]. For long-term storage, these were maintained in nutrient broth (NB) supplemented with 30% glycerol at −80 °C.

### 2.3. In Vitro Antagonistic Activity of Bacillus Strains against Bacterial and Fungal Phytopathogens

All of the *Bacillus* strains were evaluated in vitro for their antifungal and antibacterial antagonistic activity against multiple phytopathogens. Antifungal activity was checked by a dual-culture assay on Waksman agar (WA) [32,33]. Briefly, a 5 mm diameter of the fungal mycelial plug was positioned upside down at the center of the WA plate (90 mm). Three pre-sterilized filter papers (4 mm) were kept equidistantly, 2.5 cm apart from the plug, making angles of 120° among them. Two filter papers were then impregnated with 5 μL of overnight-cultivated bacterial cultures, and the remaining filter paper was impregnated with 5 μL of sterile water as the control. Plates were sealed with Parafilm^®^ M (Pechiney, Neenah, WI, USA) and incubated at 25 °C. When pathogen hyphae were grown close to bacterial isolates or over control, the degrees of inhibition (if any) by the antagonist were recorded at different angles as the growth inhibition zones (GIZs) in diameter (mm). Results are expressed as mean GIZs. Each experiment was repeated three times with at least three replicates.

Antibacterial activity was examined using the agar diffusion method on NA. After 24 h of incubation in a shaker (200 rpm) at 28 °C, a 5 mL suspension of the bacterial pathogen was mixed with 150 mL of melted NA (≤50 °C) and dispensed into plates. NA plates were permitted to solidify, followed by placing three pre-sterilized filter papers. The remaining protocol was followed as described above. Following 48 h of incubation at 28 °C, plates were stained with Gram’s iodine solution (2 g of potassium iodide (KI) and 1 g of iodine crystals per 300 mL of MQ water) for about 5 min to obtain a more distinct and clear GIZs [34]. Antibacterial activity was observed by the diameter of GIZs (mm) (if any) at different angles around the isolates. Results are expressed as mean GIZs. Each experiment was repeated three times with at least three replicates.

### 2.4. Identification of Potential Antagonists

Selected bacterial strains were identified using 16S rRNA and *gyr*B gene sequencing analyses. The genomic DNA of the selected antagonists was isolated using the TIANamp Bacteria DNA Kit (Tiangen Biotech. (Beijing) Co., Ltd., Beijing, China), considering the manufacturer’s instructions.

The partial nucleotide sequence of the 16S rRNA gene from the total genomic DNA of the isolate was amplified by the polymerase chain reaction (PCR) using universal primers: 27F (5’-AGAGTTTGATCCTGGCTCAG-3’) and 1492R (5’-GGTTACCTTGTTACGACTT-3’) [35]. The reaction mixture contained 5 μL of 10× *Ex Taq* Buffer (20 mM Mg^2+^plus), 4 μL of dNTP Mixture (2.5 mM each), 0.5 μL of *TaKaRa Ex Taq* (5 units/µL), 4 μL of MgCl_2_ (2.5 mM/L), 1 μL of genomic DNA, 2.5 μL (10 µM) of each of the primers, and sterile water made up to 50 μL. Amplification of PCR reaction was performed in a Bio-Rad S1000™ Thermal Cycler (Bio-Rad Lab., Inc., Hercules, CA, USA) using an initial denaturation step at 98 °C for 20 s, and subsequently 34 cycles of denaturing at 98 °C for 10 s, annealing at 50 °C for 30 s and extension at 72 °C for 1 min, followed by a final extension at 72 °C for 10 min. The amplified products were separated on a 1% agarose gel in TAE (40 mM Tris-acetate, 1 mM EDTA) at 100V for 1 h. The amplified bands were eluted from the agarose and purified using the E.Z.N.A.^®^ Gel Extraction Kit (Omega Bio-tek, Inc., Norcross, GA, USA) following the manufacturer’s instructions.

For *gyr*B gene sequencing, the PCR amplification of the DNA gyrase subunit B (*gyr*B) gene from the total genomic DNA of the isolate was carried out using universal primers: UP1F (5’-GAAGTCATCATGACCGTTCTGCAYGCNGGNGGNAARTTYGA-3’) and UP2R (5’-AGCAGGGTACGGATGTGCGAGCCRTCNACRTCNGCRTCNGTCAT-3’) [36]. The reaction mixture contained 5 μL of 10× *Ex Taq* Buffer (20 mM Mg^2+^plus), 4 μL of dNTP Mixture (2.5 mM each), 0.5 μL of *TaKaRa Ex Taq* (5 units/µL), 4 μL of MgCl_2_ (2.5 mM/L), 1 μL of genomic DNA, 2.5 μL (10 µM) of each of the primers, and sterile water made up to 50 μL. Amplification of PCR reaction was performed in a Bio-Rad S1000™ Thermal Cycler using an initial denaturation step at 98 °C for 15 s, and subsequently, 30 cycles of denaturing at 98 °C for 10 s, annealing at 62 °C for 30 s, and extension at 72 °C for 1 min, followed by a final extension at 72 °C for 8 min. Separation of the amplified products and elution of the amplified bands were performed as described for 16S rRNA gene analysis.

The purified PCR products of 16S rRNA and *gyr*B genes were sent to Springen, Biotech. Company (Shanghai, China) for sequencing. The obtained sequences of 16S rRNA gene of each strain were subjected to identification using EzTaxon-server (https://www.ezbiocloud.net/, accessed on 12 October 2022) and standard BLAST search at the National Center for Biotechnology Information (NCBI) for identification. For *gyr*B gene sequences, a standard BLAST search tool was used on NCBI. Furthermore, phylogenetic trees were constructed based on the sequences of closely related published-type strains and the partial sequences of bacterial antagonists according to the reported method [37] using MEGA 7 (MAC version 7.0.26) [38].

### 2.5. Effect of Bacillus *spp.* on the Growth of Rice Seedlings

The effect of selected antagonistic strains of *Bacillus* spp. on the growth of rice seedlings was evaluated in vitro using the standard roll-towel method [39,40]. Healthy rice seeds (cv. Kyou818) were surface-disinfected with 70% ethanol for 1 min followed by further disinfection with 1.65% sodium hypochlorite (NaOCl) solution for 20 min. The seeds were rinsed five times for 1 min each with double-distilled water (ddH_2_O). Bacterial cells of the overnight-grown culture of the antagonists were pelleted by centrifugation at 8000 *g* and 4 °C for 10 min and resuspended in the same volume of sterile water, adjusting the final concentration to 10^7^ CFU/mL. The seeds were soaked in antagonist suspension for 2 h followed by blotting with sterile filter paper at room temperature. Here, we used 120 mm of diameter sterile filter papers as wet blotters. Seven seeds per filter paper were arranged in a straight line at equal distances. Each filter paper was rolled, placed vertically into 350 mL of a plastic cup, and incubated in the growth chamber at 28 °C for 8 days. Adequate water was regularly given into the cups 1 cm below the arrangement of the seeds. Seeds, soaked in ddH_2_O, were served as the control (CK). The experiment was repeated twice; each treatment had five replications with three seedlings per replicate. The germination (%) [41] and vigor index (VI) [39] were calculated using the following formulae:

Percent germination = number of strongly germinated seeds/total number of seeds tested × 100;

Vigor index = percent germination × seedling length (shoot length + root length).

### 2.6. Suppression of BB of Rice by Potential Bacillus *spp.*

Two selected antagonistic strains, FA12 and FA26, and a well-known biocontrol strain FZB42, were further evaluated for their suppression ability against BB under greenhouse conditions. Surface disinfection of rice seeds and bacterial inoculation were carried out as described above. After bacterial inoculation, blotted seeds were placed evenly on wet filter paper in a sterile Petri dish (90 mm), and filter paper was impregnated with 5 mL of ddH_2_O. Plates were sealed with parafilm and incubated in the dark in the growth chamber at 25 °C for 3 days. Seeds soaked in sterile water were used as the control. Pre-germinated seeds were then planted in the boxes (21.5 cm × 31.5 cm) containing autoclaved peaty-soil (Fertile Soil Inc., Heilongjiang, China) and shifted to the greenhouse. The soil was autoclaved twice, each at 121 °C for 30 min at 15 psi. After three weeks of germination, seedlings were transplanted into nondraining 1 L pots containing autoclaved peaty-soil and further incubated in the greenhouse. The greenhouse conditions were maintained at 28 °C/25 °C for day⁄night, respectively, with 30% humidity. Adequate water was given before and after the transplantation of seedlings at regular intervals.

#### BB Inoculation and Evaluation

The BB pathogen, *Xoo*, was grown on NA plates for 3 days at 28 °C. A fresh single colony of *Xoo* was then subcultured into 20 mL of NB and shaken at 200 rpm and 28 °C for 24 h. The cell suspension was adjusted to 10^9^ CFU/mL and used as a pathogen inoculum. At the maximum tillering stage (8–10 leaves/plant), plants were sprayed with the suspension of each antagonistic strain (10^7^ CFU/mL) or ddH_2_O as control. On the third day of post-treatment, 3/4 middle leaves per treated plant were inoculated with *Xoo* suspension (10^8^ CFU/mL) by the leaf-clipping method [42]. Briefly, the flame-sterilized scissor tips were dipped into the *Xoo* suspension and clipped the leaf tip (approximately 4–5 cm) off. The inoculated cups were maintained with constant humidity and temperature in the growth chamber for at least 2 days and returned to the greenhouse. The experiment was designed in a completely randomized block and repeated thrice. Each treatment had five replications containing three plants per replicate. After 14 days of post-inoculation of *Xoo*, the lengths of the lesion area and the entire leaf area were measured [7]. Disease severity and suppression efficacy were calculated using the following formulae:

Disease severity = lesion area/entire leaf area × 100;

Suppression efficacy = {(disease severity of control–disease severity of treatment)/disease severity of control} × 100.

### 2.7. Bacillus Strains Colonization of Rice Roots

The colonization of the strains was determined in a separate greenhouse experiment. *Bacillus* strains were first checked for their antibiotic resistance and found tolerant to rifampicin (50 mg L^−1^) and chloramphenicol (10 mg L^−1^) when grown onto LB supplemented with antibiotics. The bacterial colonization capacities of FA12, FA26, and FZB42 strains on rice roots were then evaluated by rice seed treatments at a concentration of 10^7^ CFU/mL, followed by a reported protocol with some modifications [26]. Fourteen days after seed inoculations, three rhizosphere samples consisting of the whole root system with tightly adhering soil from each treatment carefully collected from the individual pots. One gram of root samples was soaked in 9 mL of sterile saline and shaken at 200 rpm for 30 min to harvest bacterial cells from the rhizosphere soil and the rhizoplane. Following serial dilutions, the cell suspension was plated on LB medium supplemented with antibiotics as mentioned above after heat shocking at 65 °C for 10 min. After incubation at 37 °C for several days, colonies were counted and expressed as log CFU per g of rhizospheric soil.

### 2.8. Analysis of Lipopeptides by MALDI-TOF MS

To demonstrate the lipopeptides spectrum of *Bacillus* spp. by MALDI-TOF MS, the strains, FA12 and FA26, were grown in Landy medium [43] at 28 °C for 38 h and were prepared according to our previous report [44]. Briefly, the cells were removed by centrifugation (8000 rpm for 25 min at 4 °C) from the surfactant-containing medium. The crude extract was precipitated from the supernatant by adding 6 N HCl to obtain a pH of 2.0. The acid precipitates were retrieved by centrifugation (8000 rpm for 15 min at 4 °C) and were extracted through methanol; the extract was neutralized immediately to avoid the formation of methyl esters. The extracts were examined by MALDI-TOF MS as described previously [45]. The mass spectra were noted on a Bruker Daltonik Reflex MALDI-TOF instrument with a 337 nm nitrogen laser for desorption and ionization. α-Cyano-4-hydroxycinnamic acid was used as the matrix.

### 2.9. Data Analysis

The results were examined by the analysis of variance (ANOVA) with Tukey’s Kramer honestly significant difference (HSD) multiple range test (*p* < 0.05) using JMP^®^ software version 14.0 (SAS Institute Inc., Cary, NC, USA) for MAC.

## 3. Results and Discussion

### 3.1. In Vitro Broad-Range Antagonistic Activity of Bacillus Strains

**Figure 1:**
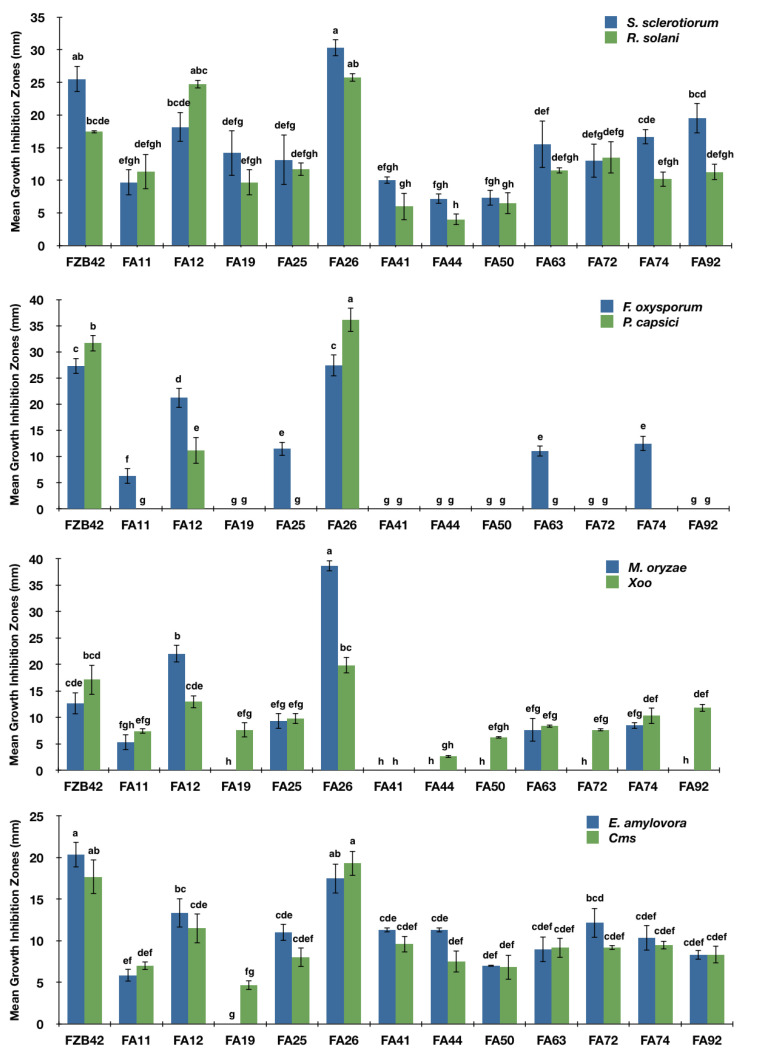
The mean growth inhibition zones (GIZs) (mm) of the selected bacterial isolates against multiple phytopathogens. GIZs were clear halos surrounding the antagonists. Error bars indicate standard deviations of the means. Different small letters above error bars indicate significance among them.

**Figure 2:**
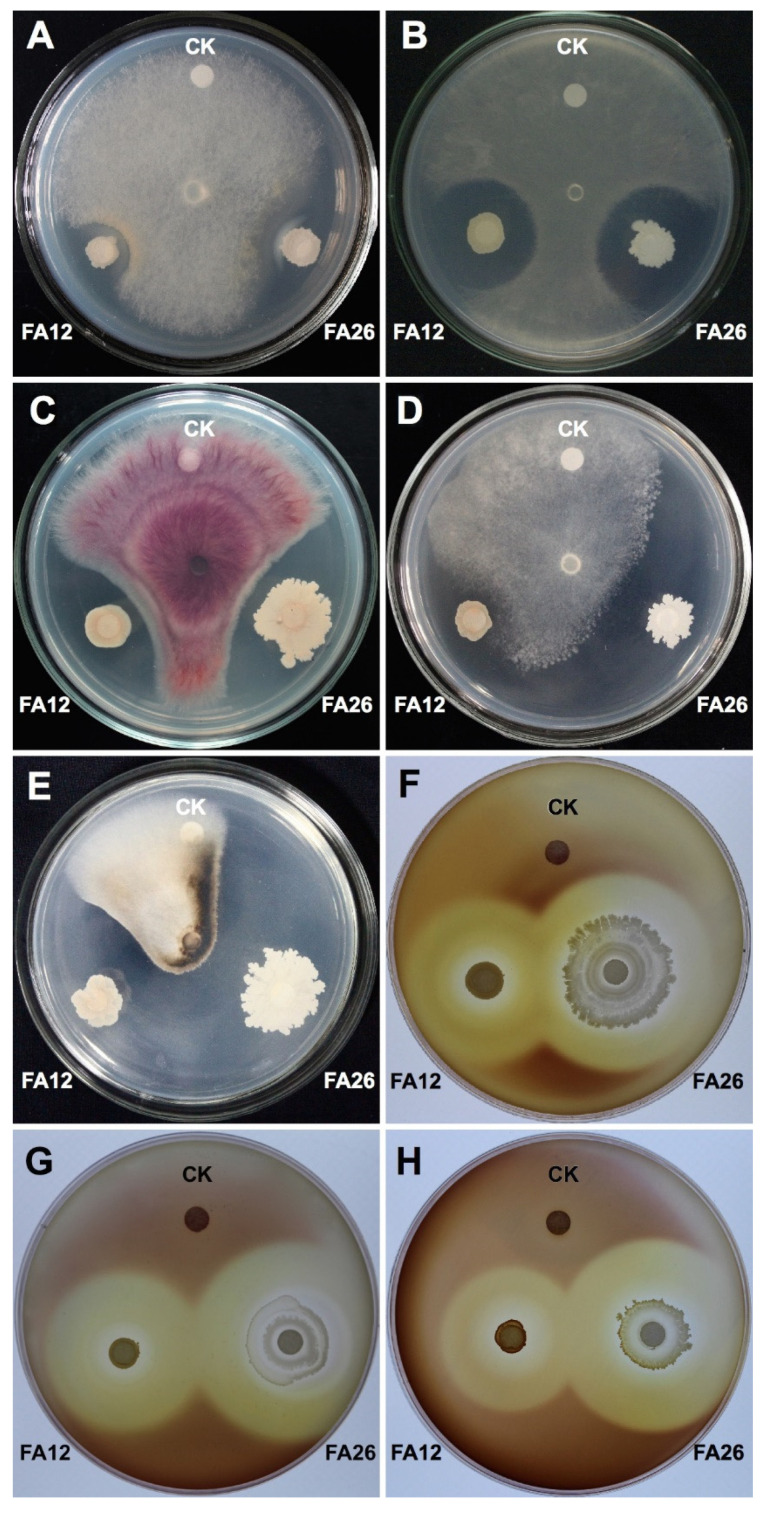
In vitro broad-range antagonistic activity of the selected strains, FA12 and FA26, against *S. sclerotiorum* (**A**), *R. solani* (**B**), *F. oxysporum* (**C**), *P. capsici* (**D**), *M. oryzae* (**E**), *Xoo* (**F**), *E. amylovora* (**G**), and *Cms* (**H**). Gram’s iodine was used to rinse the plates to obtain more distinct antibacterial GIZs around the antagonists (**F**–**H**).

The mechanism of plant disease reduction is generally believed to be due to antagonism among BCAs and phytopathogens [14]. Developing new BCAs using microbial antagonists against plant diseases requires an efficient screening strategy. In this study, we screened many bacterial isolates for their broad-range antagonistic activity against multiple phytopathogens. A total of 12 out of 141 isolates were initially selected against four phytopathogens, which were further evaluated against four more phytopathogens, including the BB pathogen, *Xoo*. The results show that each of the twelve isolates antagonized at least two of the phytopathogens, *S. sclerotiorum* and *R. solani*, shown in Figure 1. However, the isolates FA12 and FA26, as well as the reference strain FZB42, exhibited broad-range antagonism toward all test phytopathogens, and the inhibition was much greater than other antagonists (Figure 1 and Figure 2). In addition, FA26 produced larger GIZs than FZB42 against test phytopathogens, except for *Xoo* and *Cms*. Based on these results, FA12 and FA26 were selected as potential antagonists for further study. Although many bacterial strains exhibit in vitro antagonistic activity towards target pathogens, only about 1% control plant diseases in greenhouses, and even fewer show correlating properties under field conditions [1]. One of the major reasons might be the co-existence of several other pathogens in the same field [26]. Several studies have reported initially screening large numbers of bacterial isolates against multiple phytopathogens together with target phytopathogens to achieve broader antagonism [14,26,46]. In this in vitro screening strategy, we included top-ranked fungal and bacterial phytopathogens and other test pathogens to achieve broader antagonistic activity by the isolated strains. *F. oxysporum*, *M. oryzae*, *Xoo*, and *E. amylovora* have been placed among the top ten phytopathogens based on their scientific and/or economic importance [24,25].

### 3.2. Identification of Potential Antagonists

**Figure 3:**
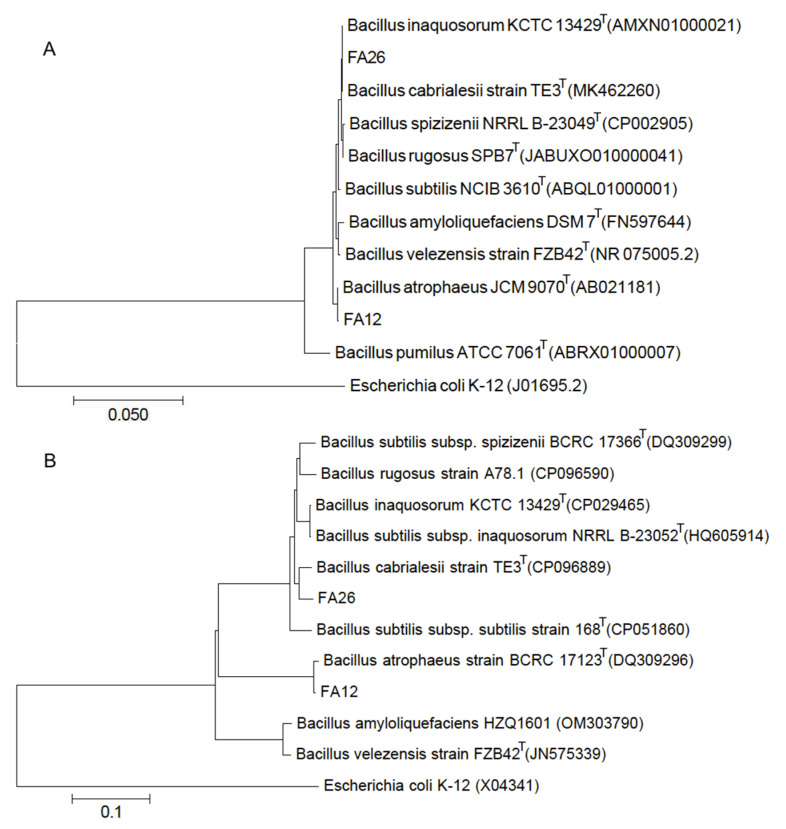
Phylogenetic trees of the selected strains of *Bacillus* spp. based on 16S rRNA and *gyr*B gene sequences. Phylogenetic trees were constructed using MEGA 7 (MAC version 7.0.26) based on partial sequences of 16S rRNA (**A**) and *gyr*B (**B**) genes of the strains and other closely related sequences obtained from the GenBank database. *Escherichia coli* K-12 was used as an out-group.

The 16S rRNA gene sequencing analysis of the strains FA12 and FA26 revealed gene lengths of 1369 bp and 1431 bp, respectively. The strains FA12 (accession no. KJ646014) and FA26 (accession no. KJ646017) showed 99.93% similarity to the *Bacillus atrophaeus* strain JCM 9070^T^ (accession no. AB021181) and 100% similarity to the *Bacillus cabrialesii* strain TE3^T^ (accession no. MK462260), respectively, by using EzTaxon-server and standard nucleotide BLAST’s tools. In addition, the sequences of 1155 bp and 1153 bp of *gyr*B genes of FA12 and FA26, respectively, further confirmed their identity and revealed that FA12 (accession no. MF116376) belongs to the *Bacillus atrophaeus* strain BCRC 17123^T^ (accession no. DQ309296) with 99.13% similarity, while FA26 (accession no. MF116377) shows 97.05% similarity with the type strain, *Bacillus cabrialesii* strain TE3^T^ (accession no. CP096889) in GenBank. Construction of phylogenetic trees based on 16S rRNA and *gyr*B gene sequences and other related sequences from NCBI GenBank revealed their close phylogenetic relationships with other *Bacillus* spp. (Figure 3).

### 3.3. Growth Promotion of Rice Seedlings by Potential Bacillus *spp.*

**Figure 4:**
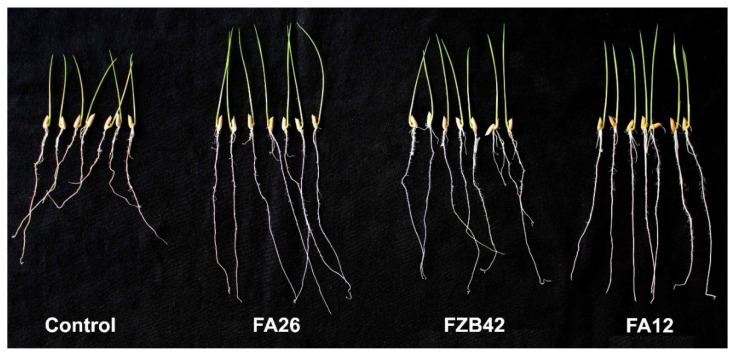
Effect of *Bacillus* spp. on the growth of rice seedlings. According to the roll-towel method, rice seeds were treated with the strains, FA26, FZB42, and FA12, for 2 h and the untreated (CK) ones were rolled with filter paper and incubated in a growth chamber. After 6 days of incubation, germination rate and seedling lengths (shoot and root) were measured before taking photos, and the vigor index was calculated.

The strains, FZB42, FA12, and FA26, used as rice seed inoculants, significantly increased the growth parameters of rice seedlings (Figure 4). Compared with the control, FA12 and FA26 increased the vigor index of rice seedlings by 78.89% and 108.70%, respectively (Table 1). Both strains also displayed better growth-promoting activity than that of reference strain FZB42 (73.73%). In addition, significantly enhanced wet and dry weights were recorded in treated seeds compared with noninoculated controls. Similar improvements in growth parameters of rice seedlings were observed when *B. subtilis* GB03 and *B. pumilus* SE34 were applied as seed treatments [47]. Growth promotion of rice seedlings using *Bacillus* spp. has been reported frequently [40,47,48], particularly for the production of several phytohormones that are important aspects of plant growth promotion [49]. This is also very important to consider that the key amongst many other factors contributing to the performance of bacterial inoculants is colonization [48]. Some researchers reported that the application of microbial antagonists using soil drenching or root dipping gives better results than seed treatment in plant growth promotion [26,50]; however, we found that seed inoculation is also an effective method of inoculation and revealed to be less laborious and time and cost-effective, and it also does not require a large amount of inoculum compared with those mentioned above. The results indicate that the selected *Bacillus* spp. used as rice seed inoculants significantly increased the plant growth parameters compared with the control. However, their growth-promoting mechanisms need to be revealed for their application in the field.

### 3.4. Suppression of BB Disease by Potential Bacillus *spp.* under Greenhouse Conditions

**Figure 5:**
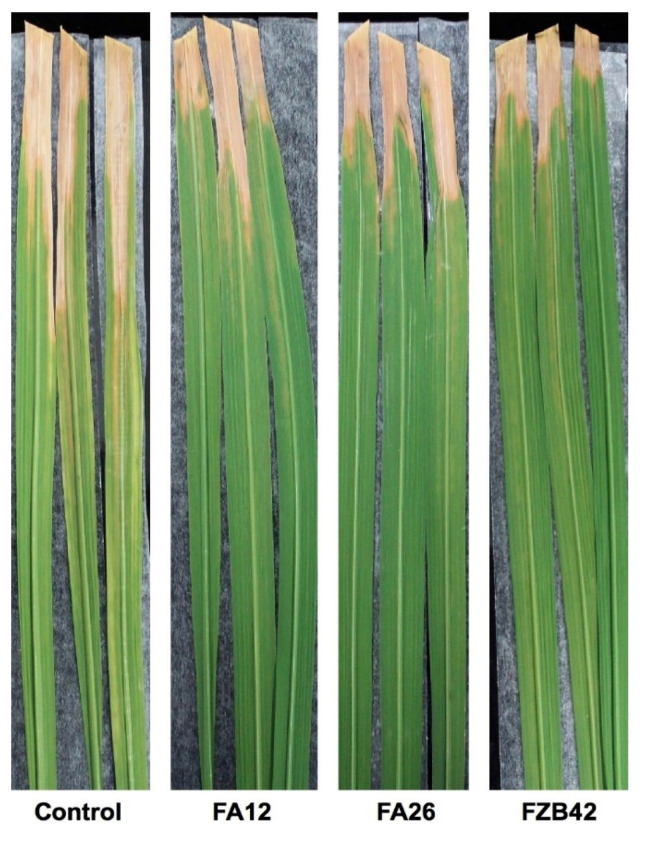
BB resistance by bacterial antagonists under greenhouse conditions. After 14 days of *Xoo* inoculation, treated leaves were detached and adhered with dual-purpose scotch tape prior to taking photos.

All three antagonists, FZB42, FA12, and FA26, were further evaluated for their suppression ability of BB disease caused by *Xoo* under greenhouse conditions. The results show that these strains significantly reduced the incidence of BB when compared with the noninoculated control (Figure 5). The lengths of lesions were significantly reduced under Bacillus-treated rice plants, exhibiting improved resistance to BB disease. Suppression efficacies of these strains ranged from 54.70–68.44% (Table 1). The reference strain, FZB42, showed slightly better suppression efficacy than FA12 and FA26. Certain strains of *Bacillus* spp. and *Pseudomonas* spp. have already been utilized as BCAs against BB and showed promising effects on the resistance of BB under greenhouse conditions [40]. Several *Bacillus* strains have been known as biocontrol bacteria that safeguard the plants through induced systemic resistance (ISR) [47]. In addition, local root colonization by beneficial microbes is also sufficient to induce ISR against a broad range of attackers [51,52]. Our results fully support the phenomenon that the antibacterial activity against *Xoo* could be associated with the detection of iturin lipopeptide by *B. atrophaeus* strain FA12, as explained later.

Researchers have frequently documented the application of species of the genus *Bacillus* against phytopathogens. However, the strains belonging to the specie *B. atrophaeus* are less acknowledged to possess biocontrol potential than those of *B. subtilis* and *B. velezensis*, or the majority of the research is focused on fungal phytopathogens. Recently, the *B. atrophaeus* strain DM6120 has shown good biocontrol potential towards strawberry anthracnose by suppressing the disease severity up to 94.44% and 88.88% when applied through soil drenching and foliar spray methods, respectively [53]. In addition, prior application of *Pseudomonas* *fluorescens* as seed inoculant has induced several host defence mechanisms [54]. Our results support the role of *Bacillus* spp., particularly in suppressing BB disease by introducing new potential antagonists such as FA12 and FA26. However, applying these strains under natural conditions is required where many other phytopathogens may encounter them and challenge their biocontrol potential against BB.

### 3.5. Colonization of FZB42, FA12, and FA26 on Rice Roots

The colonization capacity of FZB42, FA12, and FA26 on rice roots was also determined (Table 1). The results reveal that after the seed treatment, all these *Bacillus* strains were able to colonize the rice roots at up to 10^5^ CFU/g of the rhizosphere sample. The application of rhizobacteria as seed treatment has proved to be a beneficial component in integrated plant disease protection by enhancing defence-related gene expressions in treated rice plants [55], thereby playing an important role in eliciting ISR. For example, the colonization of *P. aeruginosa* 7NSK2 on rice roots induced their defence capacity against *M. oryzae* [56]. Several studies suggested that rhizocompetence is an important part of the development of efficient BCAs [26,57,58]. Our results, in line with previous studies, reveal that FA12 and FA26 had significant levels of rhizocompetence, which could have contributed to the enhanced systemic resistance against BB, which is an added advantage for practical agriculture. Apart from their activity against BB pathogen, these bacteria have shown good potential to promote rice growth.

### 3.6. Identification of Antimicrobial Lipopeptides from Bacillus *spp.*

**Figure 6:**
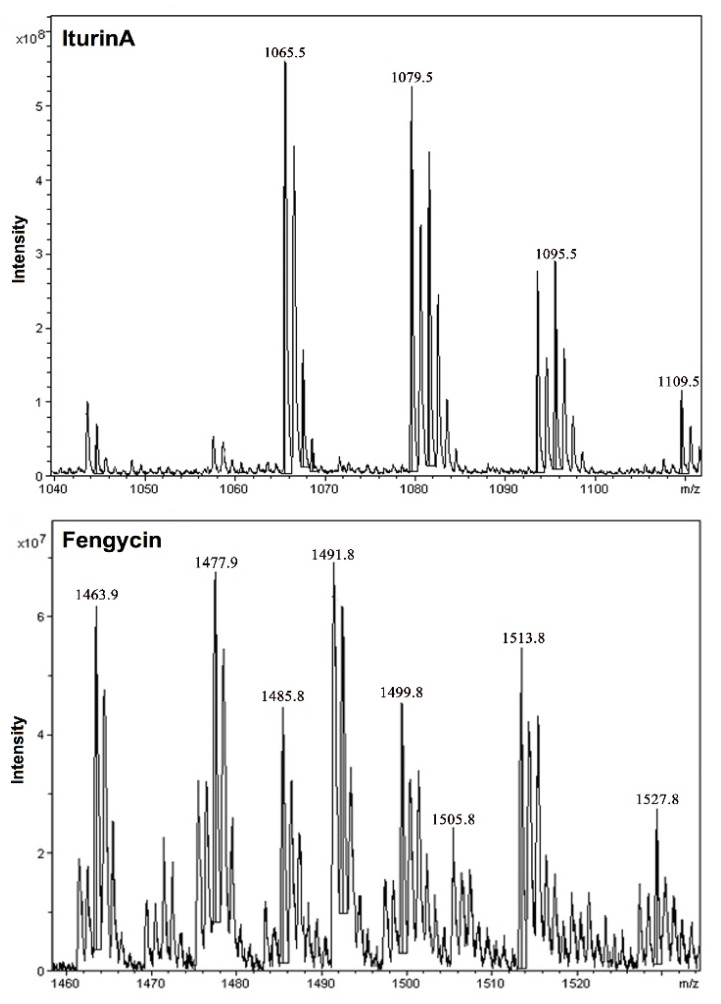
MALDI-TOF mass spectra from *Bacillus* spp. strains with the *m*/*z* range 1040–1540. *m*/*z* represent their corresponding lipopeptides.

*Bacillus* spp. has been recognized to produce broad-spectrum antimicrobial lipopeptides [44,59,60]. MALDI-TOF MS analysis was used for rapid detection of the lipopeptides produced by the *Bacillus* spp. strains FA12 and FA26. The results show that both FA12 and FA26 could produce fengycin with molecular ion peaks (M + H)^+^ for C_14_-C_17_ fengycin at *m*/*z* 1463.9, 1477.9, 1491.8, and 1505.8 and ion peaks (M + Na)^+^ for C_14_-C_17_ fengycin at *m*/*z* 1485.8, 1499.8, 1513.8, and 1529.8 (Table 2 and Figure 6). In addition, FA12 could also produce the iturin A belonging to the iturin family. The ion peaks (M + Na)^+^ for C_14_-C_15_ iturin A at *m*/*z* were 1065.5 and 1079.5, respectively, and ion peaks (M + K)^+^ for C_15_-C_16_ iturin A at *m*/*z* were 1095.5 and 1109.5, respectively. All of these molecules added with the same ion had a 14 Da difference in molecular weights, suggesting the presence of varied lengths of fatty acid chains within fengycin and iturin A (CH2 = 14 Da). These results were identical to other reports [44,60]. The detected lipopeptides from *Bacillus* spp. have shown promising biological activities, including the induction of defence-related pathways [35,44,61]. While the presence of fengycin in all members of the *B. subtilis* species complex is well-known, the occurrence of iturin in *B. atrophaeus* and closely related species are not well-documented. However, the presence of iturin and surfactin genes in the *B. atrophaeus* strain DM1620 in a recent study might be associated with its antifungal activity against *Colletotrichum nymphaeae* [53]. Previously, antibacterial activity against *X. campestris *pv. *cucurbitae* and *Pseudomonas carotovorum* subsp. *carotovorum* has also been associated with iturin lipopeptides from *Bacillus* strains [62]. Our findings support the research of Zeriouh and his colleagues [62] that the production of iturin A by *B. atrophaeus* FA12 could be associated with some of its antibacterial activities by stimulating enhanced systemic resistance against BB disease. These results may contribute to biotechnological applications, particularly developing new BCAs in ecological agriculture.

## 4. Conclusions

In this study, among several strains, *Bacillus atrophaeus* FA12 and *B. cabrialesii* FA26 were selected as broad-range antagonists and applied as seed inoculants for their suppression of BB. Both strains showed good potential in the growth promotion of rice seedlings and suppression of BB disease under greenhouse conditions and expressed the ability of root colonization. MALDI-TOF MS analysis further revealed the production of antimicrobial lipopeptides by these antagonists, which could be associated with some of their antibacterial activities by enhancing systemic resistance in rice seedlings against BB disease. Overall, the results of this study suggest that FA12 and FA26 are effective BCAs against BB under greenhouse conditions; therefore, they could be tested in formulated inoculations under natural agronomical conditions.

## Figures and Tables

**Table 1:** Effect of *Bacillus* spp. on growth parameters of rice seedlings, suppression of BB under greenhouse conditions, and colonization capacity on rice roots.

Treatments	Germination (%)	Seedling Length (cm)		Vigor Index		Wet Weight (mg)	Dry Weight (mg)	Disease Incidence (%)	Suppression Efficacy (%)	CFU/g Rhizospheric Soil Sample^*^
		Shoot	Root		Relative Increase (%)					
FZB42	95	5.7 ± 0.79b	10.1 ± 1.43a	1501.0c	73.73	768b	510b	10.38 ± 2.76b	68.44a	(2.2 ± 1.3) × 10^5^a
FA12	92	5.9 ± 0.57b	10.9 ± 0.74a	1545.6b	78.89	892a	617a	13.24 ± 1.77b	59.74b	(2.0 ± 0.5) × 10^5^a
FA26	98	6.7 ± 0.26a	11.7 ± 0.80a	1803.2a	108.70	834ab	639a	14.90 ± 1.38b	54.70c	(1.7 ± 1.6) × 10^5^a
Control	72	4.4 ± 0.20c	7.6 ± 1.80b	864.0d	–	459c	262c	32.89 ± 2.14a	–	–

Values are the mean of at least three replications (mean ± SD). Means in the same column followed by different letters are significantly different according to Tukey’s Kramer HSD multiple range test at *p * < 0.05. ^*^ Rice seeds were soaked in the suspensions of FZB42, FA12, and FA26 at a concentration of 10^7^ CFU/ml or in sterile water as the control for 2 h.

**Table 2:** Lipopeptides products of *Bacillus* spp. strains detected by MALDI-TOF-MS.

Mass Peaks (*m*/*z*)	Assignment
(1) IturinA (strain: FA12)	
1065.5 1079.5/1095.5 1109.5	C14-IturinA [M + Na]^+^ C15-IturinA [M + Na, K]^+^ C16-IturinA [M + K]^+^
(2) Fengycin (strains: FA12; FA26)	
1463.9/1485.8	C14-fengycin [M + H, Na]^+^
1477.9/1499.8	C15-fengycin [M + H, Na]^+^
1491.8/1513.8	C16-fengycin [M + H, Na]^+^
1505.8/1529.8	C17-fengycin [M + H, Na]^+^

## Data Availability

Not applicable.
